# Effect of New Zealand Sujon blackcurrant on cardiovascular responses during cycling in triathletes

**DOI:** 10.1186/1550-2783-11-S1-P11

**Published:** 2014-12-01

**Authors:** Mark ET Willems, Stephen D Myers, Matthew D Cook, Mandy L Gault

**Affiliations:** 1 Department of Sport and Exercise Sciences, University of Chichester, United Kingdom

## Background

Anthocyanin is a component known to induce vasorelaxation and vasodilation in rat aortic rings [1] and is present in high amounts in New Zealand Sujon blackcurrant. During typing work in humans, an activity of low intensity, peripheral blood flow was increased by blackcurrant intake [2]. It is not known whether anthocyanin would affect the cardiovascular responses at different exercise intensities. We examined the effect of 1-week Sujon blackcurrant powder supplementation on cardiovascular responses at low, moderate and high intensities of trained triathletes.

## Methods

Ten healthy triathletes with >3 years experience (5 men and 5 women; mean±SD: age: 40±5 years, height: 173±6 cm, body mass: 69±9 kg, BMI: 23±2, BF%: 19±4%, VO_2_max: 49±7 mL kg^-1^ min^-1^, maximum power: 293±68 W) volunteered. Participants were tested following 7 days of Sujon blackcurrant powder (S, 6g/day) or placebo (P) intake, administered following a double-blind, crossover, randomized design with a wash-out period of 4 weeks. Cardiovascular function (Portapres® Model 2, Finapres Medical Systems BV, Amsterdam, The Netherlands) was recorded during an incremental cycling protocol (4 min stages with 2 min recovery, start power 50 W with 30 W increments). Stages representing low (i.e. 40% VO_2_max), moderate (i.e. 60%) and high (i.e. 80%) intensity were analysed for responses averaged for the last minute. Paired two-tailed t-tests were used for analysis with significance accepted at p<.05. Consent to publish the results was obtained from all participants.

## Results

At each intensity, there were no differences in systolic BP (40% - P: 183±29, S: 195±31, p=.13; 60% - P: 196±35, S: 195±31, p=.40; 80% - P: 215±33, S: 220±33 mmHg, p=.50), diastolic BP (40% - P: 82±14, S: 88±17, p=.09; 60% - P: 87±14, S: 88±17, p=.39; 80% - P: 97±14, S: 101±19 mmHg, p=.36), heart rate (40% - P: 100±9, S: 101±10, p=.62; 60% - P: 126±12, S: 125±11, p=.85; 80% - P: 152±13, S: 154±11 beats min^-1^, p=.56), stroke volume (40% - P: 100±17, S: 96±24, p=.47; 60% - P: 94±20, S: 88±26, p=.53; 80% - P: 88±18, S: 88±28 mL, p=.98), cardiac output (40% - P: 10.0±1.8, S: 9.8±2.4, p=.78; 60% - P: 11.8±2.8, S: 11.6±3.1, p=.90; 80% - P: 13.3±2.6, S: 13.4±4.1 L min^-1^, p=.93), and total peripheral resistance (40% - P: 11.6±4.3, S: 13.2±5.6, p=.28; 60% - P: 10.7±4.3, S: 11.7±6.3, p=.60; 80% - P: 10.2±3.3, S: 11.3±5.5 mmHg L^-1^ min^-1^, p=.34).

## Conclusion

Previous studies indicated that anthocyanin intake may have performance-enhancing effects resulting from high-intensity training [3] and increases peripheral blood flow at very low intensity [2]. The cardiovascular responses at low, moderate and high intensity cycling in trained triathletes athletes were unaffected by one week intake of New Zealand Sujon blackcurrant powder. It is concluded that New Zealand Sujon blackcurrant does not have adverse cardiovascular effects during exercise in trained triathletes.
